# RETRACTION: Risk Factors and Healing Factors for Pharyngocutaneous Fistula after Total Laryngectomy for Laryngeal Cancer: An Epidemiological Study

**DOI:** 10.1111/iwj.70148

**Published:** 2024-12-05

## Abstract

**RETRACTION:** TaiY. , LiuF. , LiuT. , MaJ. , QinL. , JiY. , DaiH. , WangG. , MaL. , and ZangY. , “Risk Factors and Healing Factors for Pharyngocutaneous Fistula after Total Laryngectomy for Laryngeal Cancer: An Epidemiological Study,” International Wound Journal 21, no. 4 (2024): e14706, 10.1111/iwj.14706.38660912 PMC11044006

The above article, published online on 25 April 2024, in Wiley Online Library (http://onlinelibrary.wiley.com/), has been retracted by agreement between the journal Editor in Chief, Professor Keith Harding; and John Wiley & Sons Ltd. Following an investigation by the publisher, all parties have concluded that this article was accepted solely on the basis of a compromised peer review process. In addition, the authors did not disclose the details regarding the ethical approval and patient consent for the study. The editors have therefore decided to retract the article. The authors disagree with the retraction.

**RETRACTION:** Y. Tai , F. Liu , T. Liu , J. Ma , L. Qin , Y. Ji , H. Dai , G. Wang , L. Ma , and Y. Zang , “Risk Factors and Healing Factors for Pharyngocutaneous Fistula after Total Laryngectomy for Laryngeal Cancer: An Epidemiological Study,” International Wound Journal 21, no. 4 (2024): e14706, 10.1111/iwj.14706.38660912 PMC11044006

The above article, published online on 25 April 2024, in Wiley Online Library (http://onlinelibrary.wiley.com/), has been retracted by agreement between the journal Editor in Chief, Professor Keith Harding; and John Wiley & Sons Ltd. Following an investigation by the publisher, all parties have concluded that this article was accepted solely on the basis of a compromised peer review process. In addition, the authors did not disclose the details regarding the ethical approval and patient consent for the study. The editors have therefore decided to retract the article. The authors disagree with the retraction.

